# Improving the performance of an anionic MOF by counter cation replacement as electrode material in a full cell setup of a potassium ion capacitor†

**DOI:** 10.1039/d3ra01746j

**Published:** 2023-04-19

**Authors:** Chalita Aphirakaramwong, Oluseun Akintola, Christian T. Plass, Montree Sawangphruk, Winfried Plass, Andrea Balducci

**Affiliations:** a Institut für Technische Chemie und Umweltchemie, Friedrich-Schiller-Universität Jena Jena Germany andrea.balducci@uni-jena.de; b Center of Excellence for Energy Storage Technology (CEST), Department of Chemical and Biomolecular Engineering, School of Energy Science and Engineering, Vidyasirimedhi Institute of Science and Technology Rayong 21210 Thailand; c Institut für Anorganische und Analytische Chemie, Friedrich-Schiller-Universität Jena Jena Germany sekr.plass@uni-jena.de; d Institut für Festkörperphysik, Friedrich-Schiller-Universität Jena Jena Germany; e Center for Energy and Environmental Chemistry Jena (CEEC Jena) Jena Germany

## Abstract

Potassium-based energy storage devices are attracting increasing attention as an alternative to lithium and sodium systems. In addition, metal–organic frameworks (MOFs) can be considered as promising electrode materials for this type of device due to their advantageous properties. Herein, the anionic MOF JUMP-1 and its analog with pre-loading of potassium cations, namely JUMP-1(K), were synthesized and characterized. The anionic framework of JUMP-1 is found to be extremely stable towards the exchange of the dimethylammonium cations by potassium ions. These MOFs were tested in composite electrodes in combination with conventional organic electrolytes as anode materials in a potassium-based system, including the full cell assembly of a potassium ion capacitor (KIC). The results show the significant improvement in capacity between the pristine JUMP-1 and the potassium-exchanged analog JUMP-1(K) as electrode materials. KICs containing JUMP-1(K) coupled with activated carbon (AC) display a promising stability over 4000 cycles. According to the results from these studies, the composite MOF electrode with the potassium-exchange analog JUMP-1(K) presents a promising approach, for which the electrochemical performance compared to the pristine anionic MOF is significantly enhanced.

## Abstract 2

Potassium-based energy storage devices are attracting increasing attention as an alternative to lithium and sodium systems.

## Graphical abstract

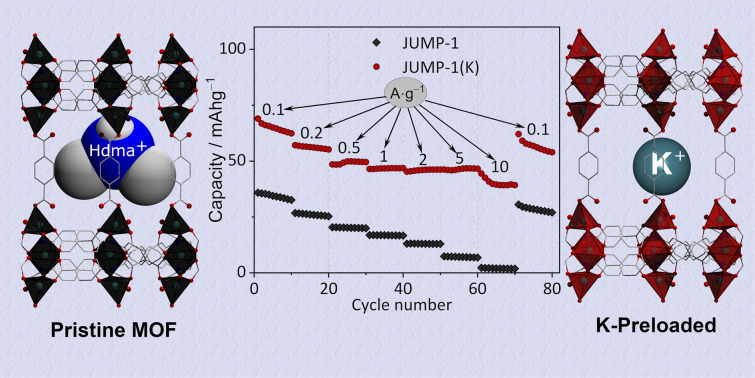

## Introduction

The development of energy storage devices based on abundant materials is nowadays considered as one of the most important aspects for the realization of an energy-efficient and sustainable society. In this context, the development of potassium-based systems has gained increasing attention due to the abundance and relatively limited cost of this metal, and the fact that it displays a reduction potential (−2.94 V *vs.* standard hydrogen electrode (SHE) for K^+^/K compared) lower than of sodium (−2.71 V *vs.* SHE for Na^+^/Na) and similar to that of lithium (−3.04 V *vs.* SHE for Li^+^/Li).^1–4^

In the last few years several materials have been investigated for the realization of potassium-based devices and, among them, metal–organic frameworks (MOFs) are presently considered one of the most interesting.^5–10^ MOFs are porous materials that belong to the polymeric coordination compounds, and the general motif of their structures consists of a polytopic organic ligand that is coordinatively linked to transition metal centers or clusters *via* donor atoms such as oxygen or nitrogen.^11–14^ Many of these compounds incorporate transition metal ions such as manganese(ii), cobalt(ii), nickel(ii), and copper(ii).^15–18^

While the vast majority of MOFs tend to be neutral, also the realization of anionic networks is possible. These compounds are indicated as anionic MOFs. The interest on this subclass of MOFs is related to the fact that their pore sizes can be tuned *via* post-synthetic cation exchange.^19–24^ Furthermore, the abundance of electronegative sites within their structure can enable the ion mobility within the channels.^25–27^ In a recent work, we investigated the use of different anionic MOFs with a pillared-layer architecture, which we indicated as JUMP-1, as anodic materials for lithium and sodium ion batteries.^28^ We have shown that the nature of the cation balancing the charge of the anionic MOF has a strong influence on the electrochemical performance of electrode containing these materials. Additionally, we have demonstrated that pre-loading the anionic MOF with the cation that is used as charge carrier in the relevant system is beneficial for the electrode performance. Considering these results, the use of designed anionic MOFs can be therefore considered as an interesting strategy to introduce these materials in energy storage devices. At this point it is worth noting that several proposals toward a possible storage mechanism have been reported for the underlying building units of the JUMP-1 system, namely the terephthalate pillars^5,7,29,30^ and the triarylamine linkers,^31^ suggesting that both organic moieties can contribute to the storage of alkali metal ions in MOFs.

In this work, we report for the first time the use of JUMP-1 and its potassium-exchanged analog, indicated as JUMP-1(K), as anodic materials for potassium-based energy storage devices. Initially, the synthesis, the morphological, and the structural properties of the anionic MOF are considered. Afterwards, the electrochemical behavior for composite electrodes containing JUMP-1 and JUMP-1(K) in combination with a potassium-based electrolyte is considered. Finally, the electrochemical performance of a potassium-ion capacitor (KIC) based on JUMP-1(K) is investigated.

## Experimental section

### Materials

4-Fluorobenzonitrile (Alfa Aesar), 4-aminobenzonitrile (Alfar Aesar), potassium nitrate, and cobalt(ii) chloride hexahydrate (Aldrich) were obtained commercially and used without further purification. All other chemicals were of AR grade.

### Methods

Simultaneous TG/DTA analyses were performed under static air atmosphere using a Netzsch STA Luxx PC analyser up to 1000 °C. The FT-IR spectra were measured on a VERTEX 70 IR spectrometer by Bruker Optics using the Specac Diamond ATR optional accessory. The elemental analyses were done on a VARIO EL III analyser. Solvothermal reactions were carried out in a 23 mL Teflon-lined acid digestion vessel from Parr Instruments, utilizing a programmable oven by Binder. The argon physisorption isotherms were measured on an Autosorb-IQ instrument from Anton Paar. Powder X-ray diffraction (PXRD) measurements were performed on a Stoe Powder Diffractometer with a Mythen 1K detector at room temperature. Measurements were done using capillary tubes while the Debye Scherrer scan mode was applied with a 2*θ* scan type. The X-ray tube was a Cu-long fine focus tube. The powdered samples were placed in a 0.5 mm glass capillary and then measured. The measurement was carried out between 2 and 50° with steps of 2.1° per 20 seconds. SEM and EDXS measurements of the composite electrodes were performed using a FEI dual beam FIB with high-resolution electron beam. The EDXS elemental maps were obtained at 15 keV.

### MOF synthesis and characterization

#### ((CH_3_)_2_NH_2_)_2_[Co_3_(ntb)_2_bdc]·2H_2_O·4DMF (JUMP-1)

JUMP-1 was synthesized under hydrothermal conditions utilizing a mixture of 4,4′,4′′-nitrilotribenzoic acid (H_3_ntb) and terephthalic acid (H_2_bdc) as linkers and cobalt(ii) chloride in DMF as previously reported.^32^ The obtained solid material was washed repeatedly with DMF to remove any unreacted ligand or metal salt. The obtained JUMP-1 was then dried under vacuum until constant weight was achieved. Elemental analysis calcd for JUMP-1 C_66_H_76_Co_3_N_8_O_22_ (*M* = 1510.15 g mol^−1^): C, 52.49; H, 5.07; N, 7.42%. Found: C, 52.35; H, 5.00; N, 7.70%. Selected IR data (ATR, cm^−1^): 1657s, 1592s, 1504w, 1385vs, 1312s, 1275s, 1171m, 1130m, 1092w, 834w, 779s, 675m.

#### K_2_[Co_3_(ntb)_2_bdc]·2H_2_O·4DMF (JUMP-1(K))

JUMP-1(K) was obtained by immersing the as-synthesized JUMP-1 in a saturated solution of KNO_3_ in DMF. During this period, the solution used for soaking was replaced every 24 h, while the sample was washed repeatedly with pure solvent each time to remove residual potassium salt. After each 24 h soaking period, a sample of the obtained material was subjected to elemental analysis after thorough drying. It was found that five cycles were required to reach the constant composition of the fully potassium-exchanged material. To ensure completeness of the exchange, two extra cycles were added to lead to a final seven days period for the exchange procedure. The potassium-exchanged material was then dried under vacuum until constant weight was obtained. Elemental analysis calcd for JUMP-1(K) C_62_H_60_Co_3_N_6_O_22_K_2_ (*M* = 1496.16 g mol^−1^): C, 49.77; H, 4.04; N, 5.62%. Found: C, 50.12; H, 3.71; N, 5.53%. Selected IR data (ATR, cm^−1^): 1667s, 1590vs, 1506s, 1387vs, 1313vs, 1274s, 1172s, 1139m, 1092w, 832w, 779s, 676m.

### Argon sorption measurements

#### MOF pretreatment

Prior to sorption measurements, JUMP-1(K) was activated using supercritical CO_2_. The drying procedure was performed using a K850 Critical Point Dryer provided by Quorum Technologies. About 20 mg of JUMP-1(K) was immersed in ethanol (10 mL) for seven days during which the solvent was refreshed daily. The supernatant was decanted off and the samples carefully transferred into the small porous pots and then into the sample holder. The drying chamber was at this point precooled to 5 °C, after which the sample was quickly transferred into the chamber and then hurriedly but carefully sealed tight. The chamber was then filled with liquid CO_2_ and stirred for 30 min. Subsequently, the stirring was stopped, and the liquid CO_2_ was allowed to slowly drain off. The chamber was re-cooled down to 5 °C and then refilled with liquid CO_2_ and this time allowed to stand for 24 h while stirring. At the end of the 24 h period, the stirrer was turned off and the chamber was once again slowly emptied and allowed to briefly stand empty. For a third time, the chamber was cooled down to 5 °C and then filled while stirring for another one hour followed by slow release. In a final run, after cooling the chamber down and then filling with liquid CO_2_, the heater was started and after 35 min, the CO_2_ was brought to supercritical conditions and maintained for a further 90 min. The gas was then slowly released while keeping the heater on to prevent freezing.

#### Measurement of isotherms

The isotherms of the activated product were measured immediately after outgassing the samples for 30 min at room temperature using argon at 87 K. Pore size distribution curves were calculated by fitting the experimental data using a non-local density functional theory (NLDFT) kernel based on adsorption models for argon on zeolites/carbon at 87 K with cylindrical pores, which was provided by QUANTACHROME Instruments (QUANTACHROME). The Brunauer–Emmett–Teller (BET) surface areas for JUMP-1(K) was determined from the adsorption data over the relative pressure range between 0.005 to 0.080 while ensuring compliance with the consistency criteria (see ESI Tables S1 and S2†).^33^

### Electrode preparation and cell assembly

#### MOF pretreatment

About 1 g of the material was immersed in ethanol (100 mL) for seven days during which the solvent was refreshed daily. After immersion, the supernatant ethanol was removed and the ethanol-MOF slurry, after the supernatant solvent left only a thin film of ethanol (to ensure sample did not dry out during transfer), was transferred into an autoclave and sealed. Liquid CO_2_ was then introduced into the autoclave (100 mL at a pressure of 60 bar) and allowed to stand 30 min after which the CO_2_ was then slowly released from the autoclave over a period of 20 min to remove any possible non-occluded ethanol from the materials. Subsequently liquid CO_2_ was reintroduced into the reactor and this time allowed to stand for 24 h. After this period, the temperature of the autoclave was raised to 40 °C to bring the CO_2_ to supercritical conditions and maintained for one hour. The gas was then slowly released over 30 min with the temperature maintained at 40 °C to prevent any cooling that might result from expansion of the gas during evaporation.

#### Preparation and cell assembly

The electrodes were prepared by mixing each of the pretreated MOF samples of JUMP-1 and JUMP-1(K) with carbon black as a conductive carbon (C-NERGY Super C65, Imerys) and polyvinyl fluoride (PVDF, Sigma Aldrich) as a binder in the ratio of 65 : 30 : 5 in the *N*-methyl-2-pyrrolidone (Sigma-Aldrich, 99.5%) solvent until obtained a homogeneous slurry. Similarly, the electrodes of activated carbon (AC, Norit) were prepared by mixing with carbon black (C-NERGY Super C65, Imerys) and carboxymethyl cellulose (CMC, Walocel) as a binder in the ratio of 90 : 5 : 5 in water. The obtained slurries were then casted using doctor blade. The prepared slurries of MOFs and activated carbon (AC) were coated by doctor blade on the copper foil with a wet film thickness of *ca.* 200 μm and the aluminum foil with a wet film thickness of *ca.* 500 μm as the current collectors, respectively. The average mass loading after drying was about 1.15 g cm^−2^ for JUMP-1, 0.81–1.02 g cm^−2^ for JUMP-1(K) and 7.05 g cm^−2^ for AC.

All the electrodes have been tested in combination with 1 M potassium hexafluorophosphate (KPF_6_) in a mixture of 1 : 1 ethylene carbonate (EC) and dimethyl carbonate (DMC). The electrolyte was prepared in a glovebox (Labmaster, 477 MBRAUN GmbH) under argon atmosphere with oxygen and water contents below 0.1 ppm. The electrolytes were stirred overnight until obtaining colorless and homogeneous solutions.

The electrochemical measurements were performed utilizing a 3-electrodes Swagelok-type cell. For the half-cell configuration, the prepared MOFs electrodes were used as working electrode (WE), while metallic potassium was employed for both the counter electrode (CE) and reference electrode (RE). For the full-cell configuration, the AC and MOFs electrodes were used as working electrode and counter electrode, respectively. Metallic potassium was used as reference electrode. Glass fiber (Whatman GF/D glass microfiber filters with 675 mm thickness, Whatman) and polymer separator (25 μm thickness, Celgard) were used as the separators. 160 μL of electrolyte were drenched into the separator, independently of its nature.

Cyclic voltammograms have been measured between 0.005 and 3 V *vs.* K/K^+^ using scan rates ranging from 0.1–100 mV s^−1^. Galvanostatic charge and discharge measurements were carried out in different current densities ranging from 0.1 to 10 A g^−1^ within the potential range of 0.005 and 3 V *vs.* K/K^+^. The cycling stabilities of the devices were tested carrying out 1000 cycles and 4000 cycles at a current density of 1 A g^−1^ for half-cell and full-cell configurations, respectively.

The electrochemical measurements have been realized utilizing a VMP multichannel potentiostatic-galvanostatic workstation (Biologic Science Instruments, VMP 3) and an Arbin potentiostatic-galvanostatic workstation (Arbin instruments, LBT21084) at the room temperature.

## Results and discussion

### MOF synthesis and characterization

**Fig. 1:**
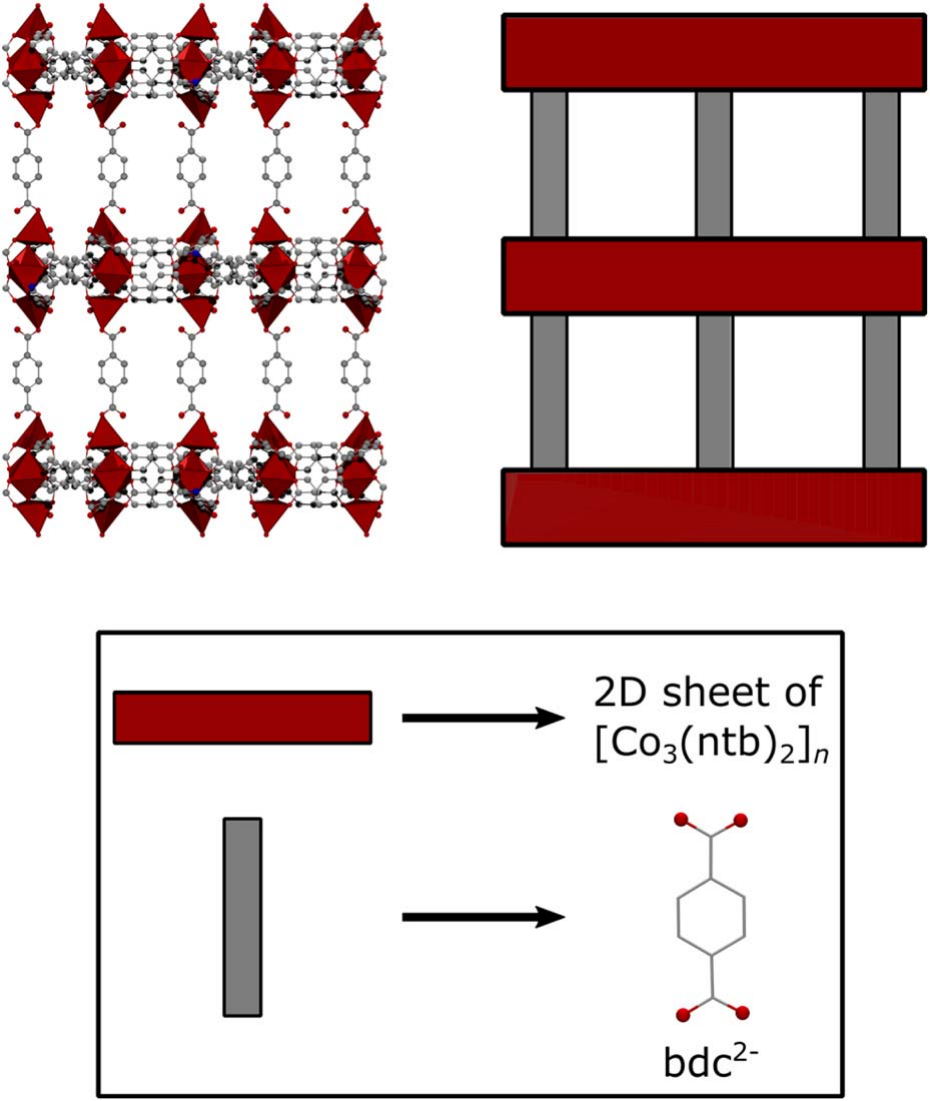
Schematic representation of the anionic network of JUMP-1.

**Fig. 2:**
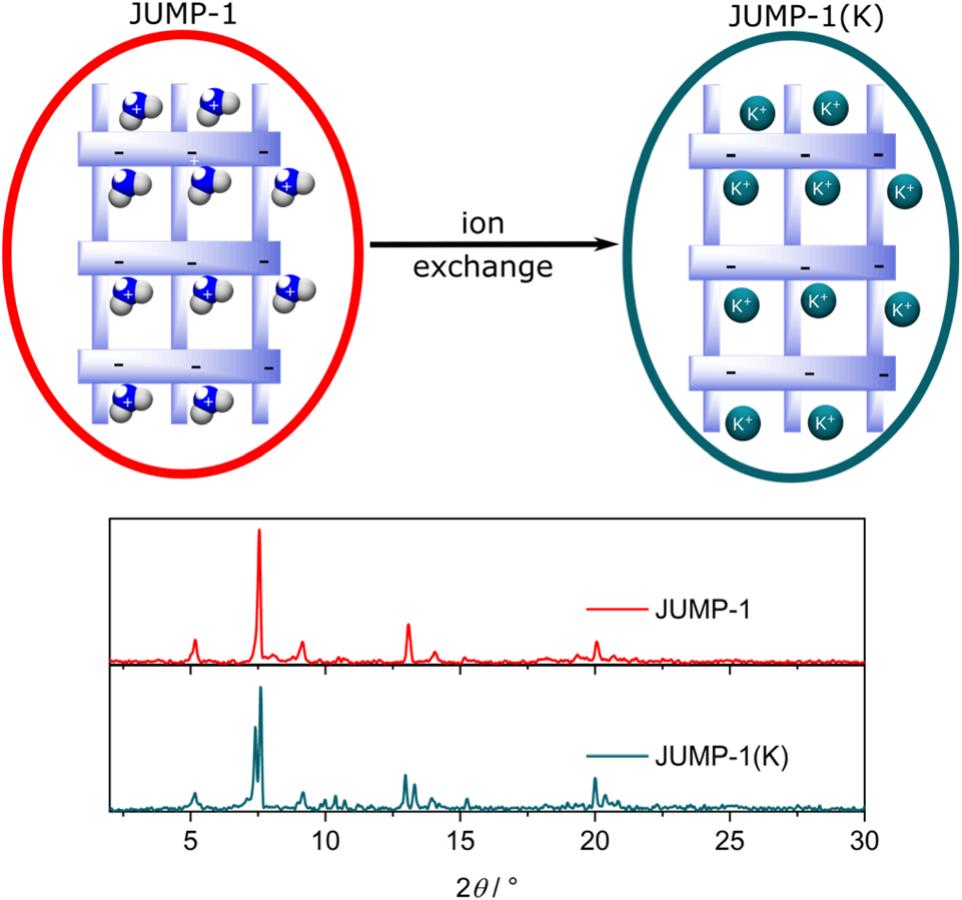
Representation of the cation exchange for JUMP-1 leading to JUMP-1(K) (top) and the corresponding powder X-ray diffraction (PXRD) diagrams before and after cation exchange.

To obtain JUMP-1, a mixture of cobalt(ii) chloride hexahydrate with 4,4′,4′′-nitrilotribenzoic acid (H_3_ntb), and terephthalic acid (H_2_bdc) were combined in dimethylformamide (DMF) solution and allowed to undergo a solvothermal reaction under autogenous pressures. JUMP-1 is composed of an anionic network comprising three cobalt(ii) ions as metal nodes together with two ntb^3−^ and one bdc^2−^ ligands as linkers, resulting in a repeating unit of [Co_3_(ntb)_2_bdc]^2−^ (see Fig. 1). The overall double negative charge of the anionic network is counterbalanced by two dimethylammonium cations resident within the pores of the framework of the MOF. These cations can be easily replaced by a simple immersion the as-prepared material in saturated DMF solution of the target ion, in this case KNO_3_. The resulting new framework is denoted as JUMP-1(K), in which the organoammonium cations have been completely replaced by potassium cations (see Fig. 2). The elemental compositions of both JUMP-1 and its potassium-exchanged analog JUMP-1(K) were determined by CHN analysis. Supplementary to this, thermogravimetry was used to confirm the replacement of the organic cation in the latter network. Clear evidence is given by the disappearance of the specific feature around 280 °C in the DTG trace characteristic for the dimethylammonium ions when present in JUMP-1 (Fig. S1†).

The stability of the network structure of the post-cation exchanged JUMP-1(K) was confirmed by powder X-ray diffraction (PXRD) measurements (see Fig. 2). The recorded diffraction patterns show minor differences between the pristine JUMP-1 and its potassium-exchanged analog JUMP-1(K). This confirms the robustness of the anionic network and the retention of its structure after potassium ion exchange. In addition, this shows that the inclusion of potassium ions is much less destructive to the network than that of sodium ions and similar to that of lithium ions (see Fig. S2†).^28^

To improve pore accessibility, we subjected the networks to solvent exchange *via* immersion in ethanol. This replaces the DMF with ethanol molecules in the pores, which facilitates drying by supercritical CO_2_ and thereby ensures better accessibility of the pores. The two methods described in the experimental section differ only in terms of the amount of material, which is related to the equipment required, and leads to the same characteristic pore properties. The resulting porosity of the potassium-exchanged analog JUMP-1(K) has been investigated by measuring the argon adsorption isotherms at 87 K for the activated material (Fig. S3†). For JUMP-1(K), this leads to a BET surface area of 258 m^2^ g^−1^ with a pore volume of 0.21 cm^3^ g^−1^ which is considerably larger than that observed for the non-exchanged JUMP-1 (see Table S1†). This is consistent with the decrease in size of the counter-cations present in the pores of the network. Moreover, the pore distribution analysis shows a marked appearance of micropores in JUMP-1(K) compared to the pristine JUMP-1 material (Fig. S3†). At this point, it should also be noted that the potassium-exchanged JUMP-1(K) exhibits comparatively much better sorption behavior than the sodium-exchanged JUMP-1(Na). This can be attributed to a significantly reduced structural stress on the network by inclusion of the potassium in contrast to sodium ions, which is evident from the corresponding powder diffractograms, which show distinct structural changes in the case of sodium but not in the case of the potassium-exchanged JUMP-1(K) (see Fig. S2†). It is therefore not unexpected that the pores in JUMP-1(K) are more accessible than in its sodium ion analog, although the trend in the ionic radii would suggest the opposite behavior.

### Electrochemical studies

**Fig. 3:**
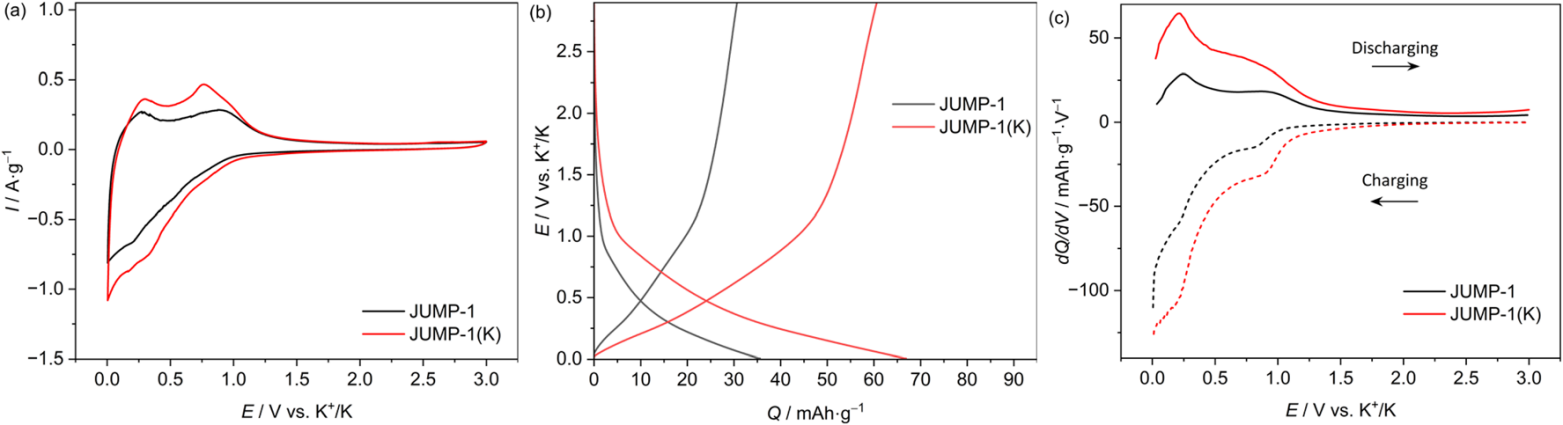
(a) CV profiles at 1 mV s^−1^, (b) galvanostatic charge/discharge profiles, and (c) differential capacity plots at 0.1 A g^−1^ of JUMP-1 and JUMP-1(K) in 1 M KPF_6_ in EC/DMC electrolytes. All data were obtained in the half-cell configuration.

Fig. 3 shows the CV profiles (carried out using a scan rate of 1 mV s^−1^) of composite electrodes containing JUMP-1 and JUMP-1(K) as the active material. As shown, in both electrodes a reversible insertion and extraction of potassium ions is taking place below 1 V *vs.* K^+^/K. However, it is important to notice that the overall amount of current density is higher in the electrode containing JUMP-1(K) and, also, that in this latter electrode the peaks associated to the insertion/extraction of potassium ions are sharper and more intense compared to those of the JUMP-1 based electrode. This difference is caused by the fact that the use of JUMP-1(K) allows the storage of a large amount of charge compared to that of JUMP-1. This is well visible in Fig. 3b, which is comparing the galvanostatic charge–discharge profiles of the two electrodes during tests carried out at 0.1 A g^−1^ within a potential range of 0.005–3 V *vs.* K/K^+^. As shown, the electrode containing JUMP-1 displays a discharge capacity of 36 mA h g^−1^, while the one containing JUMP-1(K) is 67 mA h g^−1^. As shown in the Fig. 3a, this difference is mainly caused by the ability of this latter electrode to store higher amount of charge through all the investigated potential, and especially in the region between 0.7–0.2 V *vs.* K/K^+^. This is also well visible comparing the variation of the differential capacity over the potential of the two electrodes, which is shown in Fig. 3c. The different charge storage ability of the two electrodes is clearly related to their structure, and this result confirms that the pre-loading of the anionic MOF with the cation that is used as charge carrier is an effective strategy to improve the capacity of the electrode containing these active materials.

**Fig. 4:**
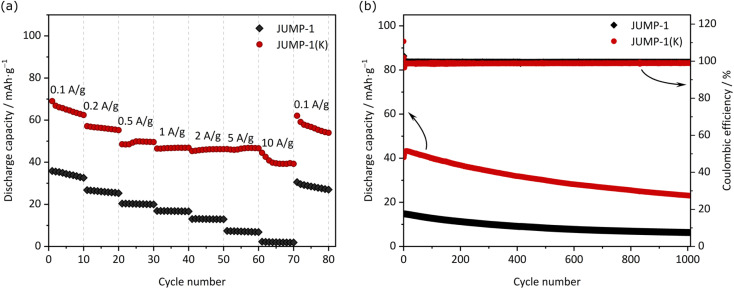
(a) Rate capabilities at different applied current densities from 0.1 to 10 A g^−1^ and (b) cycling stabilities at 1 A g^−1^ of JUMP-1 and JUMP-1(K) in the half-cell configuration within the potential range from 0.005 V to 3 V *vs.* K^+^/K.

Fig. 4a compares the capacity retention of the two investigated electrodes at different current densities. As shown, at 0.2 A g^−1^ the electrode containing JUMP-1 displays a capacity of 26 mA h g^−1^, while that containing JUMP-1(K) is 56 mA h g^−1^. When the current density is increased to 5 A g^−1^ this latter electrode displays a capacity of 46 mA h g^−1^, while the former is only able to deliver 7 mA h g^−1^. This difference is indicating that the pre-loading of anionic MOF is not only increasing the electrode capacity but is also significantly increasing the capacity retention of the electrodes during test at high current density. As shown in Fig. 4b the electrode containing JUMP-1(K) is also displaying an acceptable capacity retention over 1000 cycles. Although the capacity retention is not higher than that of the electrode containing JUMP-1, the capacity of this latter electrode is still much lower than that of the JUMP-1(K).

**Fig. 5:**
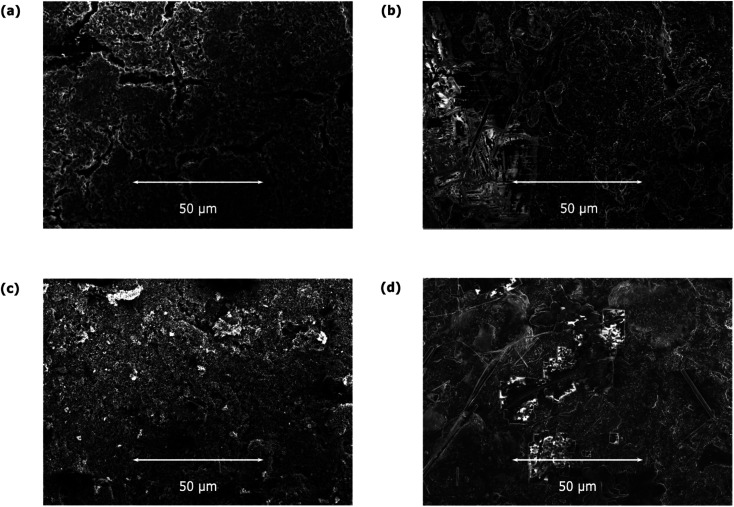
SEM images of electrodes prior to cycling for (a) JUMP-1 and (c) JUMP-1(K), and after cycling for (b) JUMP-1 and (d) JUMP-1(K) in 1 M KPF_6_ in EC/DMC electrolyte.

To gain further insight into how the morphology of the materials is affected by the electrochemical processes, we carried out SEM measurements on the prepared electrodes before and after electrochemical cycling (see Fig. 5 and S4–S7†). The obtained SEM images for JUMP-1 and JUMP-1(K) prior to cycling do not reveal any significant differences between them (Fig. 3a and c). However, post-cycling SEM images (Fig. 3b and d) show distinct changes to their surfaces. Scans taken at higher magnification (1 and 5 μm) show that the individual grains in the pre-cycle samples of JUMP-1 (Fig. S4e and f†) and JUMP-1(K) (Fig. S5e and f†) had undergone fusion after the electrochemical cycling processes (JUMP-1: Fig. S6e and f,† JUMP-1(K): Fig. S7e and f†). This feature seems to be more pronounced in the case of JUMP-1(K), which is attributed to the higher current flowing through the material compared to its non-exchanged analog JUMP-1. This is consistent with observations reported for the lithium-exchanged analog JUMP-(Li).^28^ Nonetheless, the electrodes did not reveal any obvious sign of major degradation resulting from the extended cycling, indicating that the framework is very tolerant to the insertion of potassium ions.

The compositional characteristics of the electrode materials were further addressed by additional EDXS measurements, which are shown in Fig. S8–S15.† An important feature of the electrode materials prior to their electrochemical cycling was the localization of the elemental distribution of cobalt, which was consistent with the clumps of the MOF within the composite. This was observable in both the JUMP-1 and its potassium-exchanged analog JUMP-1(K). After electrochemical cycling, the elemental distribution is much more uniform, consistent with the conclusion from the SEM images where the cycled materials showed features of fusion within the composites. Furthermore, the additional features seen in the SEM images could be conclusively identified as solid residues of the electrolyte, as they are chiefly composed of fluorine, phosphorous, and a larger proportion of potassium than the rest of the materials, especially for JUMP-1(K). Overall, the distribution of these elements in the recorded maps is consistent with what is expected for the materials both prior and subsequent to being subjected to electrochemical cycling. Additionally, the SEM images confirm the stability of the materials upon contact with the potassium ions, as already indicated by the powder diffraction data, with only minor degradation in its structure and morphology.

**Fig. 6:**
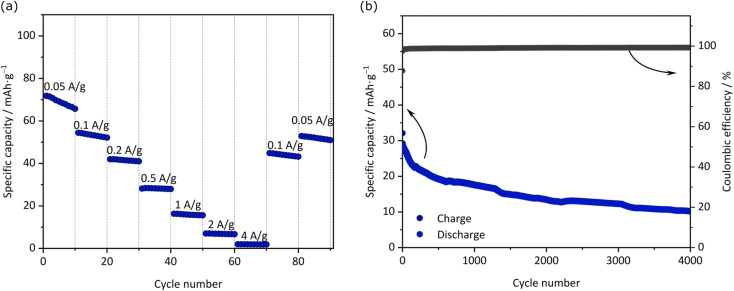
(a) Specific discharge capacity with different applied current densities and (b) cycling stability of the full-cell KIC over 4000 cycles using JUMP-K//AC at 1 A g^−1^.

In view of these results, the electrode based on JUMP-1(K) appears to feature very interesting properties. With the aim to understand its use in full cells, we realized a KIC containing an AC-based positive electrode and JUMP-1(K) as the negative electrode. Prior to being used in the full-cell, the JUMP-1(K) electrode pre-potassiated utilizing the protocol reported in literature.^34^ The electrochemical performances of the KICs are depicted in Fig. 6. At 0.05 A g^−1^ the device displays a discharge capacity of 72 mA h g^−1^, while at 0.5 A g^−1^ its capacity is 28 mA h g^−1^. When the current density is further increased, the capacity decreases significantly. Not unexpectedly, the capacity did not fully recover when going back to lower current densities, which can be attributed to (i) a non-optimized electrode balancing, (ii) the occurrence of degradation processes caused by an uncontrolled electrode drift during the cycling, or (iii) the increase of resistance. Fig. 6b shows the cycling stability of the device during 4000 cycles carried out at 1 A g^−1^. As shown, the capacity of the KIC decreases quite fast during the first 250 cycles, but thereafter the capacity fading becomes smaller and gradually stabilizes to become nearly constant by the end of the cycle process. To our knowledge, this is the first report of a full cell assembly exhibiting such a long and stable cyclic performance for a potassium-based system. The overall performance of the full cell is in good agreement with reports on other MOF-based electrodes for potassium-ion batteries, for which specific capacities of about 56 mA h g^−1^ for MIL-125(Ti)^5^ and MOF-5 (ref. 7) were obtained at current densities of 0.2 and 0.5 A g^−1^ after 2000 and 3000 cycles, respectively. A somewhat larger specific capacity (188 mA h g^−1^ at a current density 1 A g^−1^) was reported for a cobalt(ii)-based MOF with a terephthalic acid linker, however, only a performance up to 600 cycles. Considering that our JUMP-1(K)-based KIC has not been optimized, its observed stability, although not outstanding, can be considered very promising. In this context, it is also important to note that the coulombic efficiency of the charge–discharge process remains higher than 99% throughout the whole cycling process.

## Conclusions

We investigated the anionic pillared-layer metal–organic framework (MOF) JUMP-1 as a potential anodic material for the use in potassium-storage devices. JUMP-1 was modified by replacing the as-synthesized dimethylammonium cations present in the pores with potassium ions to yield the material denoted as JUMP-1(K). Further analysis showed that anionic pillared-layer framework of JUMP-1(K) does not undergo any relevant structural changes upon ion exchange in the pores. Moreover, the potassium-exchanged MOF possesses a significantly improved accessibility of the framework pores compared to the pristine JUMP-1. Tests on their performance in potassium-based energy storage devices revealed promising electrochemical performances for both MOF systems. It is interesting to note that the JUMP-1(K) system, in which the countercharge of the anionic framework is balanced by pre-exchanged potassium cations in the pores, shows a significantly higher discharge capacity compared to the pristine anionic MOF system JUMP-1, for which the framework charge is balanced by dimethylammonium cations generated during synthesis. This indicates that for JUMP 1(K), where the appropriate charge carriers are provided by pre-loading, the insertion and removal of potassium ions is facilitated during cycling. This is evident from the discharge capacity measured for JUMP-1 and JUMP-1(K) at an applied current density of 0.1 A g^−1^ with values of 36 and 67 mA h g^−1^, respectively. In addition, both half-cells utilizing JUMP-1 and JUMP-1(K) as composite electrode material provide cycling stabilities up to 1000 cycles carried out at 1 A g^−1^. Based on this promising performance of the potassium-based system, JUMP-1(K) was used as electrode material in potassium-ions hybrid capacitors. This was achieved by coupling JUMP-1(K), which was pre-loaded with potassium as negative electrode, with activated carbon (AC) as positive electrode to assemble the full-cell KIC device. The electrochemical results for this device reveal a capacity of 72 mA h g^−1^ at current densities of 0.5 A g^−1^. Its cycling stability has been tested over 4000 cycles and shows that the device is able to maintain a decent amount of capacity and display high coulombic efficiency (more than 99%) during the charge–discharge process. The results of these studies clearly show that for the anionic MOF system JUMP-1, the exchange of the cationic charge carriers in the pores of the framework for potassium ions allows a significant improvement of the electrochemical performance and represents a promising approach for the future design of electrode materials for potassium storage devices.

## Conflicts of interest

There are no conflicts to declare.

## Supplementary Material

RA-013-D3RA01746J-s001
