# Supplementary material for: Improving the performance of an anionic MOF by counter cation replacement as electrode material in a full cell setup of a potassium ion capacitor
Source: RSC Adv. 2023 Apr 19;13(18):12277–84. doi: 10.1039/d3ra01746j (PMC10113920; doi:10.1039/d3ra01746j)
Supplement: RA-013-D3RA01746J-s001 [file RA-013-D3RA01746J-s001.pdf]

Electronic Supplementary Material (ESI) for RSC Advances.
This journal is © The Royal Society of Chemistry 2023

Supplementary Material

1. Differential thermogravimetry (DTG)

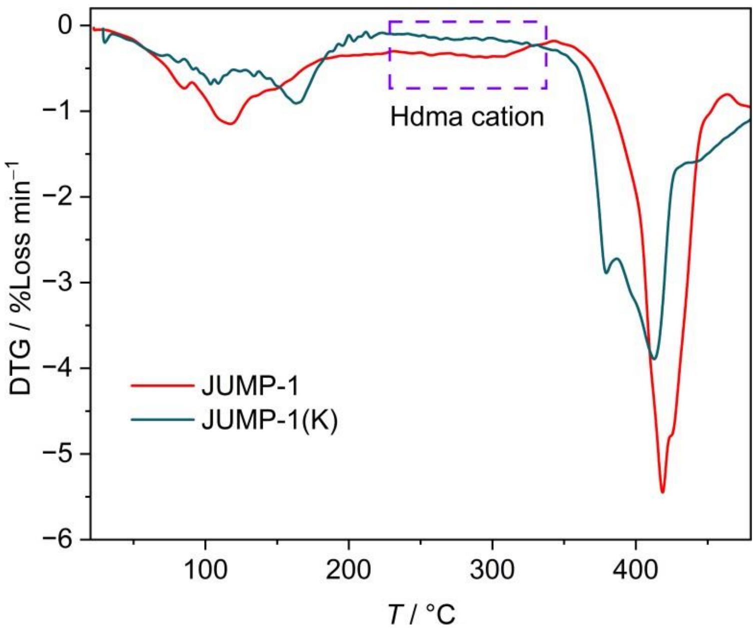

Fig. S1: DTG of the as-synthesized JUMP-1 and the potassium-exchanged JUMP 1(K) samples. Rectangular area marked with broken lines highlights the temperature range of the decomposition of the dimethylammonium (Hdma) cations indicative for their presence/absence in the framework.

2. X-ray Powder Diffraction

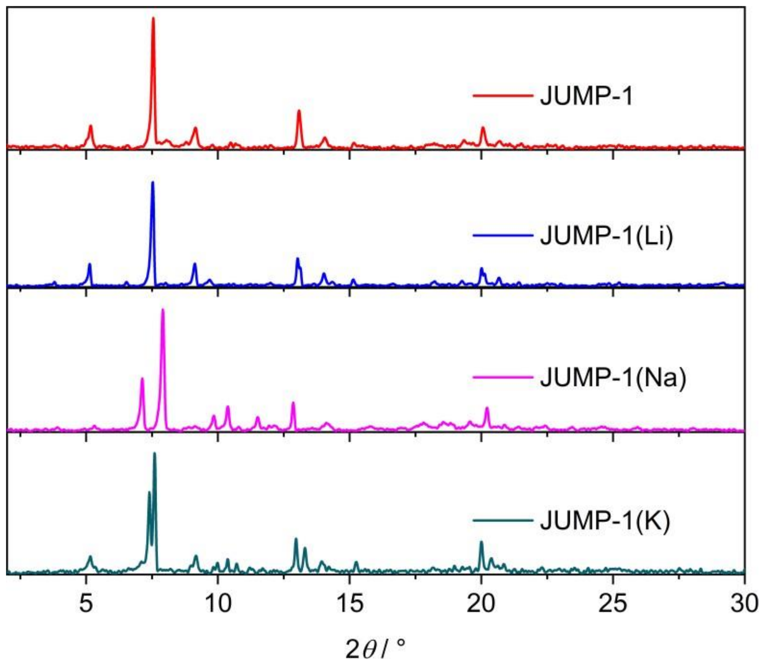

Fig. S2: Comparison of the powder X-ray diffraction (PXRD) patterns for the samples of the as-synthesized JUMP-1, lithium-exchanged JUMP-1(Li), sodium-exchanged JUMP-1(Na), and potassium-exchanged JUMP-1(K). Data for JUMP-1(Li) and JUMP-1(Na) are taken from the reference: O. Akintola, P. Gerlach, C. T. Plass, A. Balducci, W. Plass, *Front. Chem.*, 2022, **10**, 836325.

3. Sorption studies

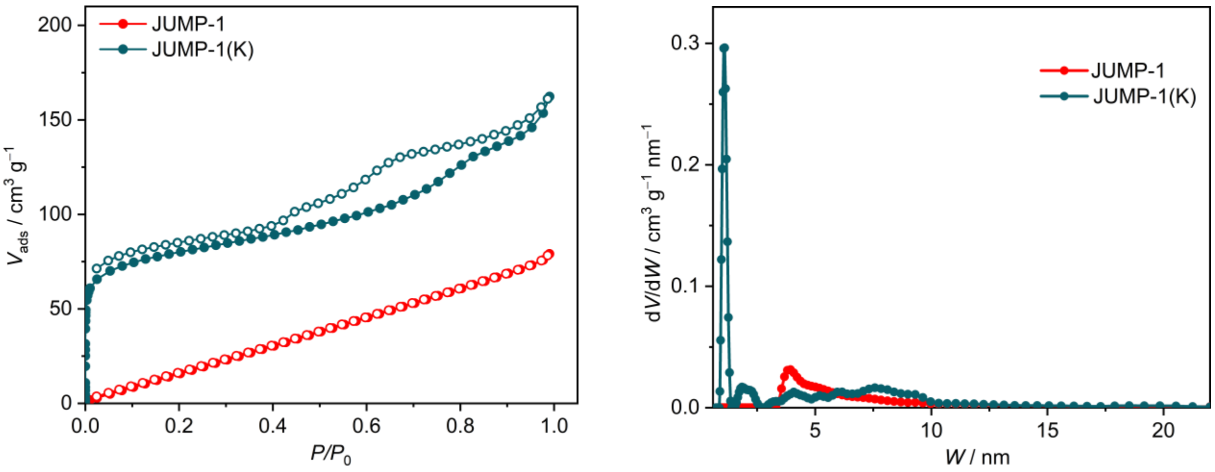

Fig. S3: Argon sorption isotherm of JUMP-1 and JUMP-1(K) (left). Pore distribution plots obtained from fitting its adsorption isotherms to NLDFT kernel (right).

Table S1: Summary of sorption data derived from argon sorption isotherms of the as-synthesized JUMP-1 and the potassium-exchanged JUMP-1(K) samples

	JUMP-1[†]	JUMP-1(K)
*a*BET ($m^2 \cdot g^{-1}$)	100	258
Pore volume (cm^3/g)	0.10	0.21
Modal pore width (nm)	3.9	1.10
Mean pore width (nm)	3.9	3.20

[†]Data taken from the reference: O. Akintola, P. Gerlach, C. T. Plass, A. Balducci, W. Plass, *Front. Chem.*, 2022, **10**, 836325.

Table S2: Consistency criteria for calculating BET surface areas for as-synthesized JUMK-1 and the potassium-exchanged JUMP-1(K) samples

	JUMP-1[†]	JUMP-1(K)
P/P_0 *range*	0.20–0.35	0.005–0.079
C	3.8	828.9
V_m (cm^3/g)	25.9	67.7
$(\sqrt{C} + 1)^{-1}$	0.34	0.034
P/P_0 (V_m)	0.34	0.038
*a*BET (m^2/g)	100	258
R	0.999	0.9999

[†]Data taken from the reference: O. Akintola, P. Gerlach, C. T. Plass, A. Balducci, W. Plass, *Front. Chem.*, 2022, **10**, 836325.

4. SEM Images

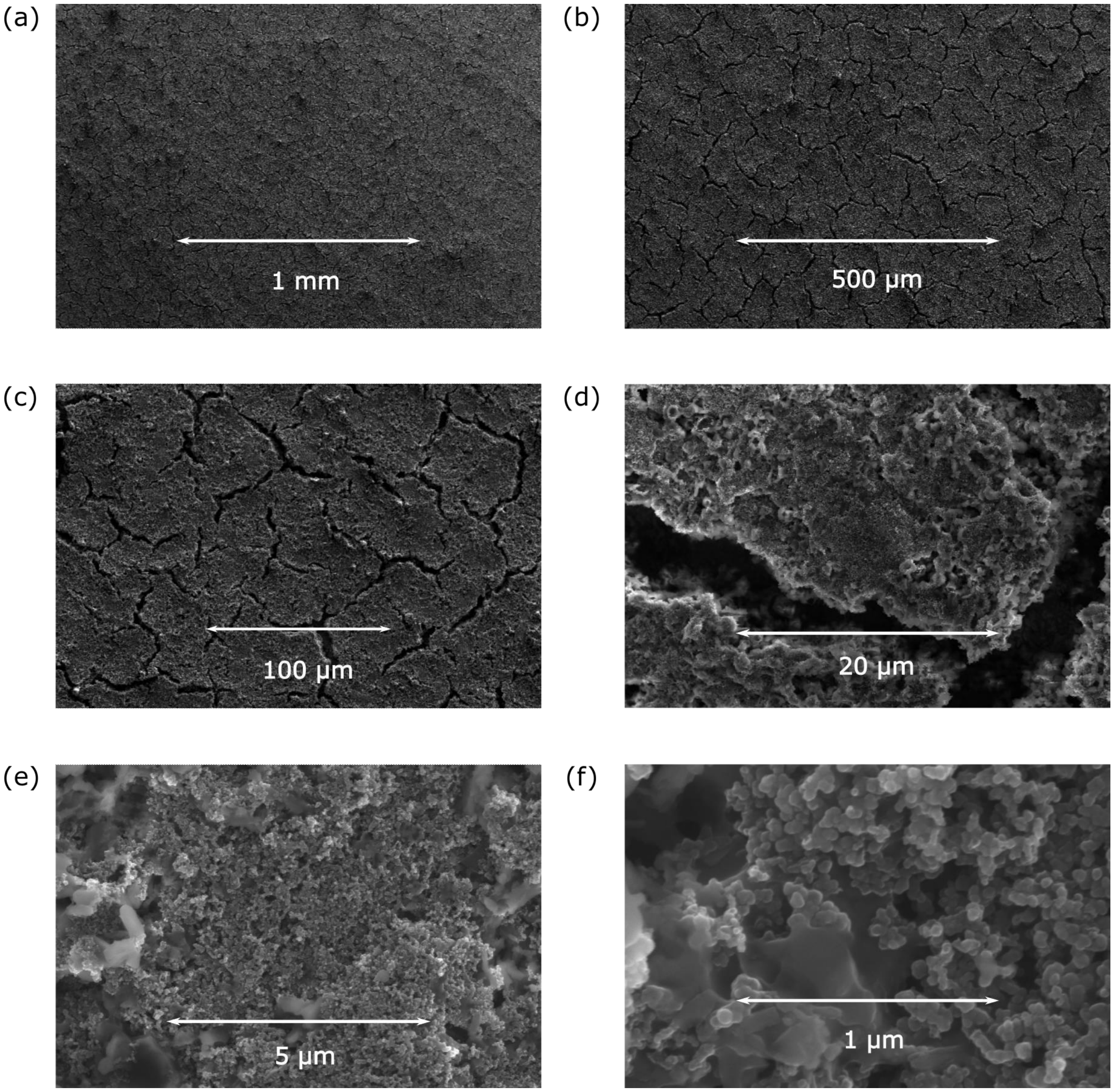

Fig. S4: SEM Images of JUMP-1 prior to electrochemical cycling.

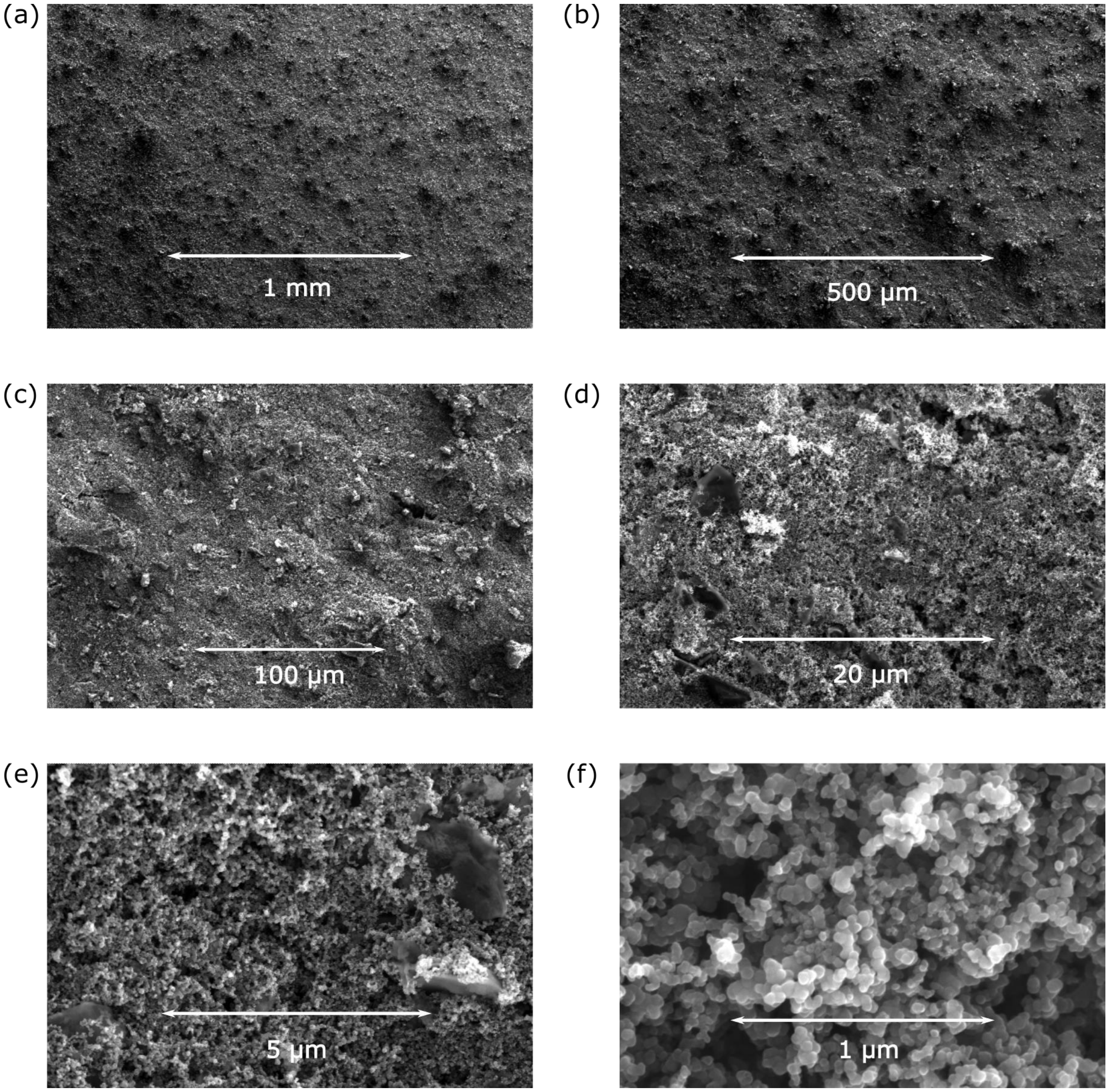

Fig. S5: SEM Images of JUMP-1(K) prior to electrochemical cycling.

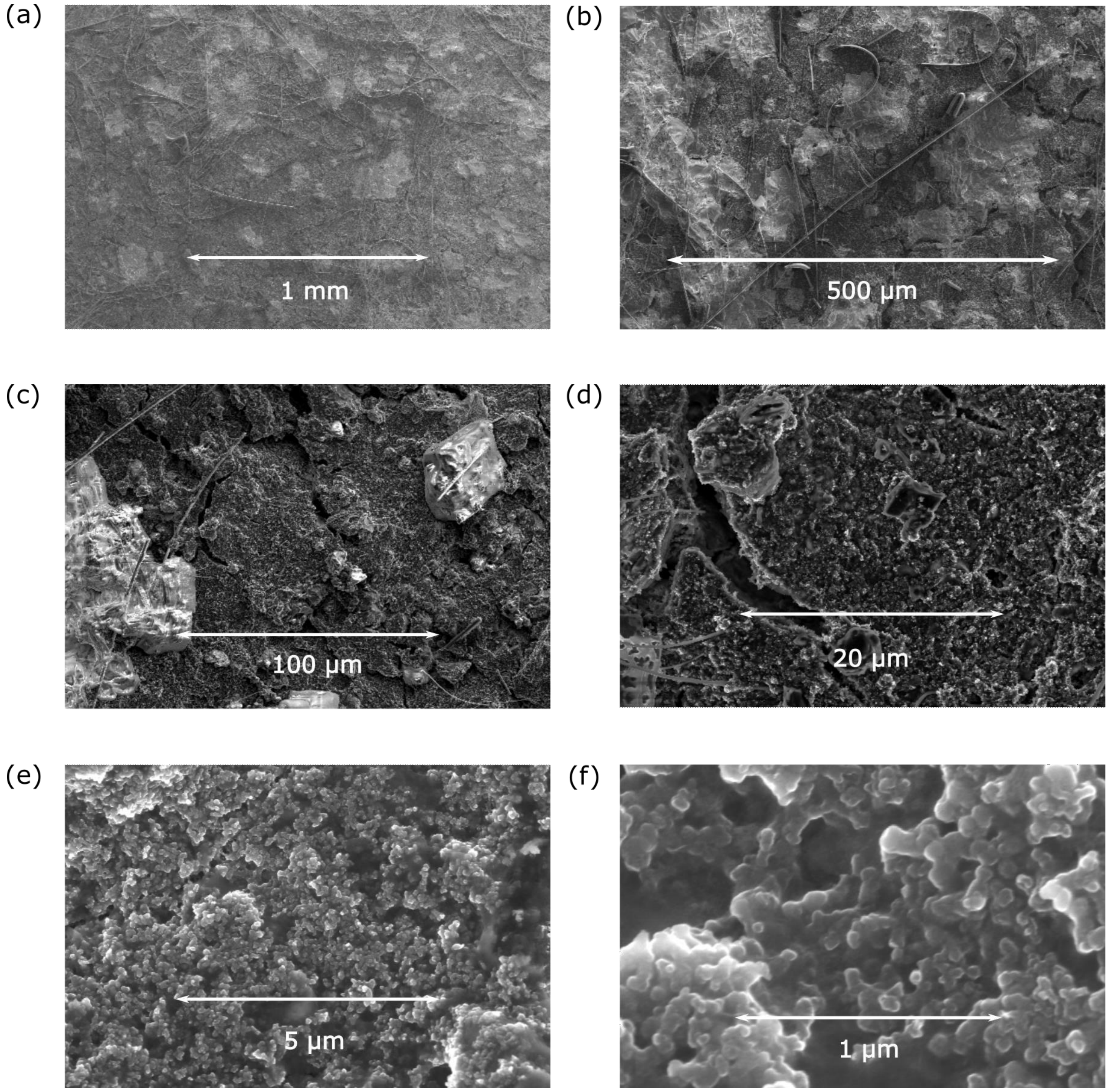

Fig. S6: SEM Images of JUMP-1 after electrochemical cycling.

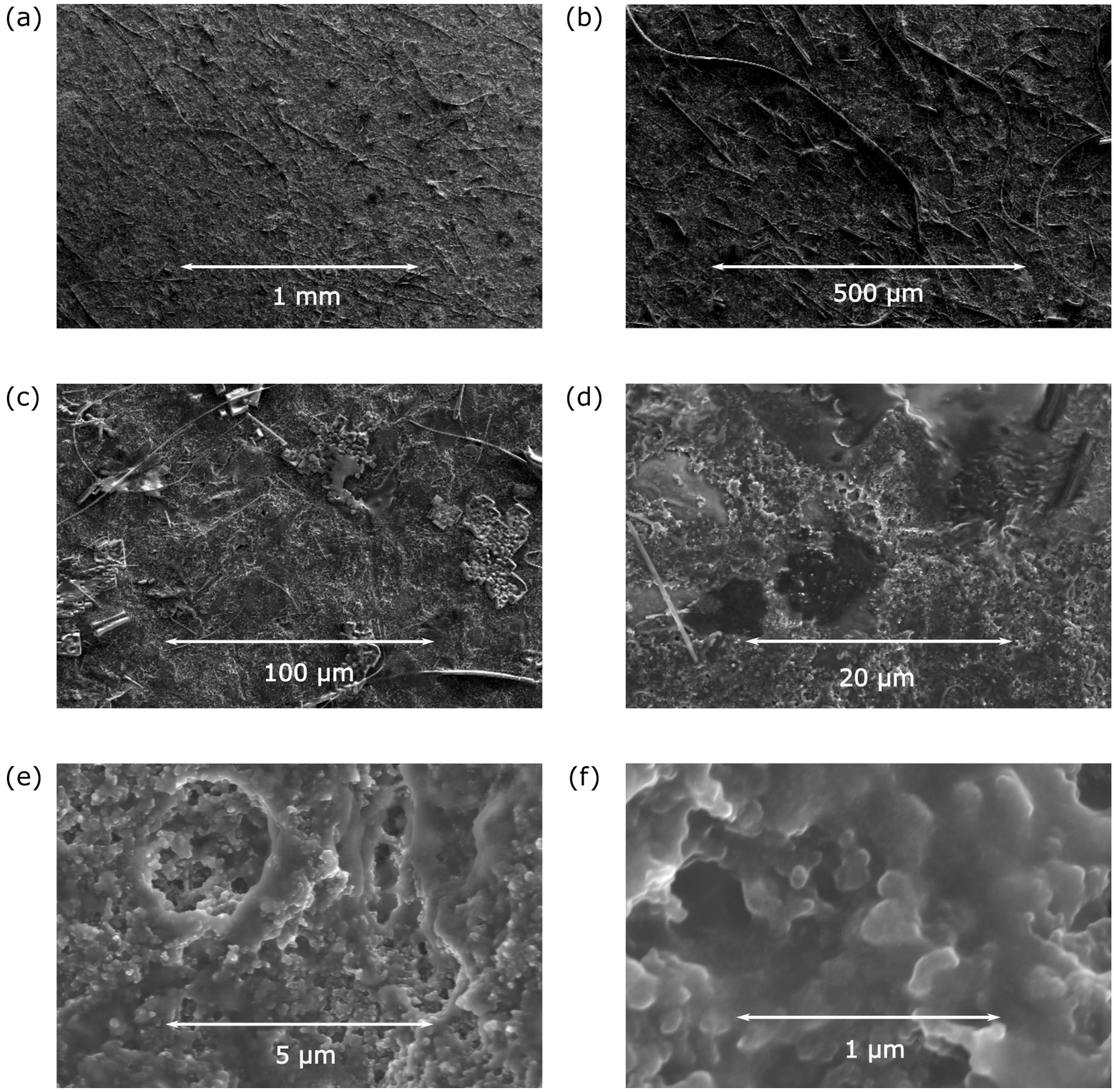

Fig. S7: SEM images acquired for JUMP-1(K) after electrochemical cycling.

5. Electron Dispersive X-ray Spectroscopy (EDXS) Mapping images

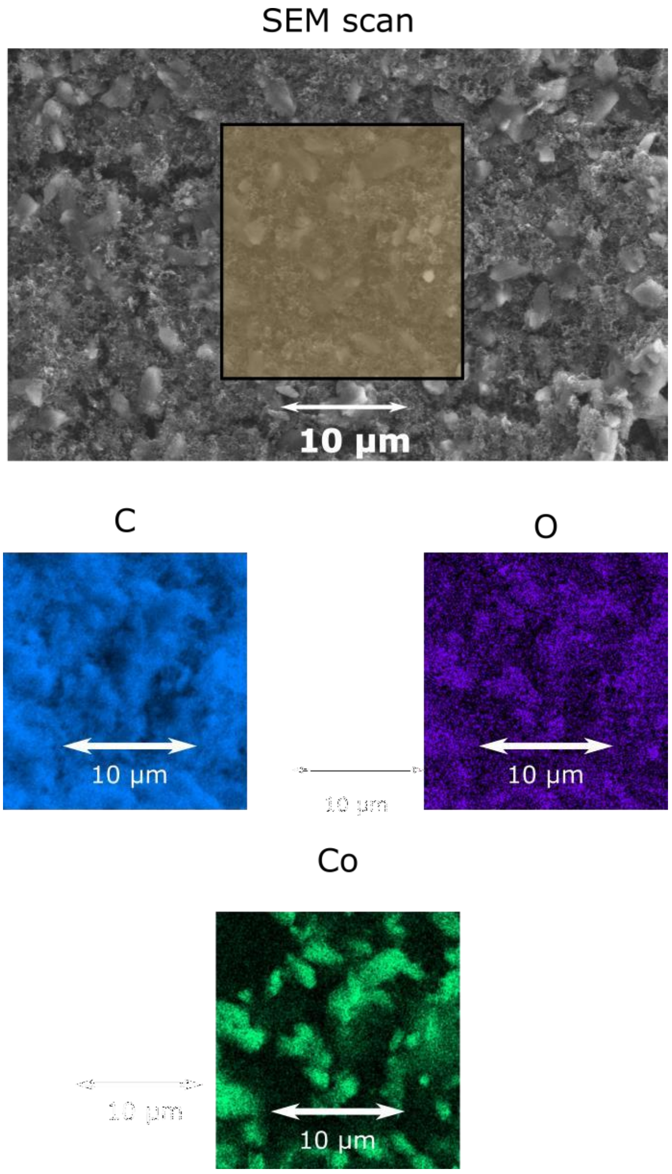

Fig. S8: EDXS intensity maps for JUMP-1 prior to electrochemical cycling at lower magnification.

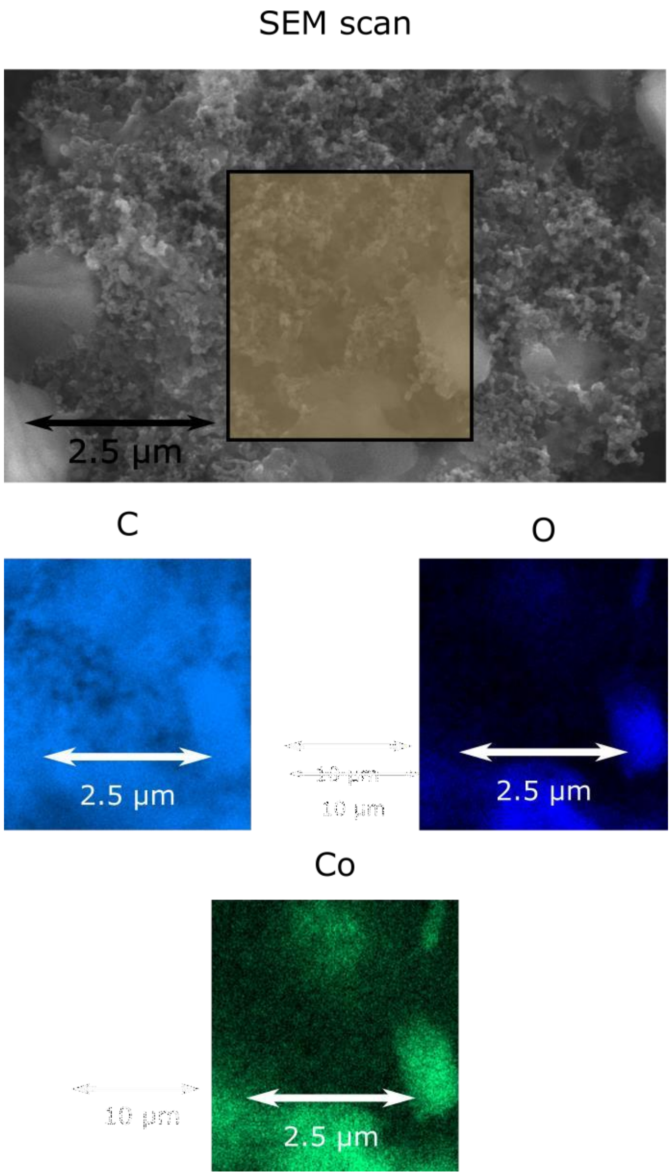

Fig. S9: EDXS intensity maps for JUMP-1 prior to electrochemical cycling at higher magnification.

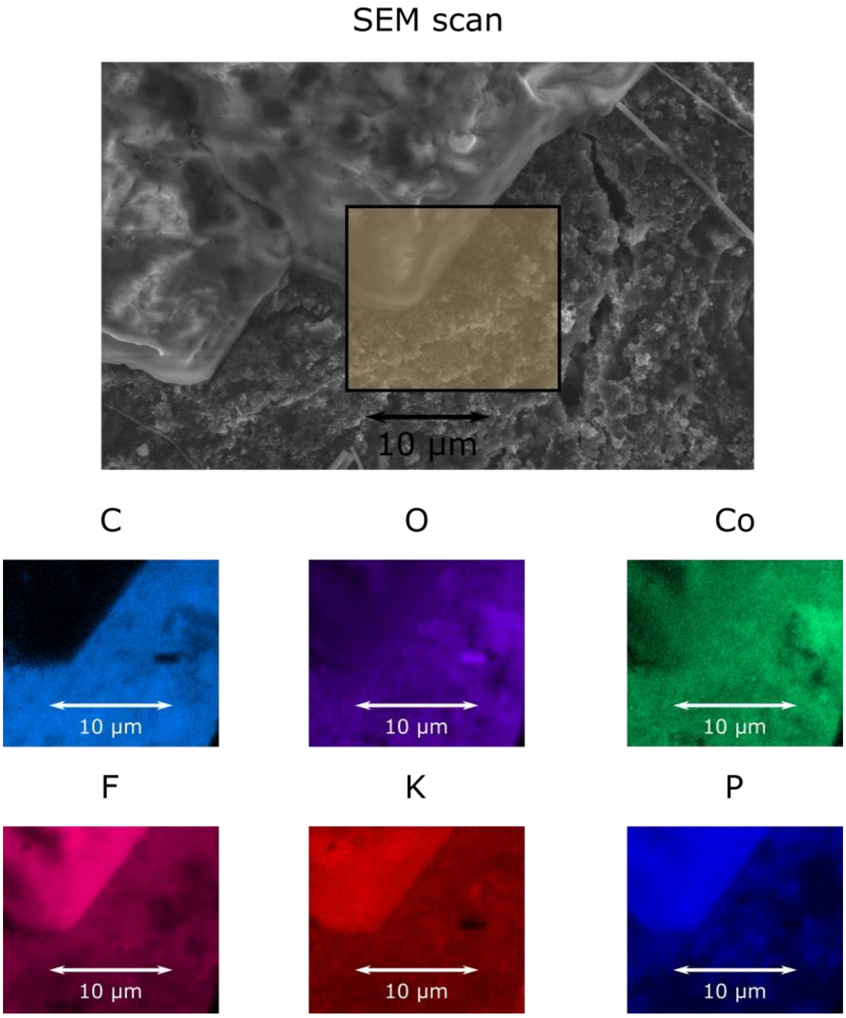

Fig. S10: EDXS intensity maps for JUMP-1 after electrochemical cycling at lower magnification.

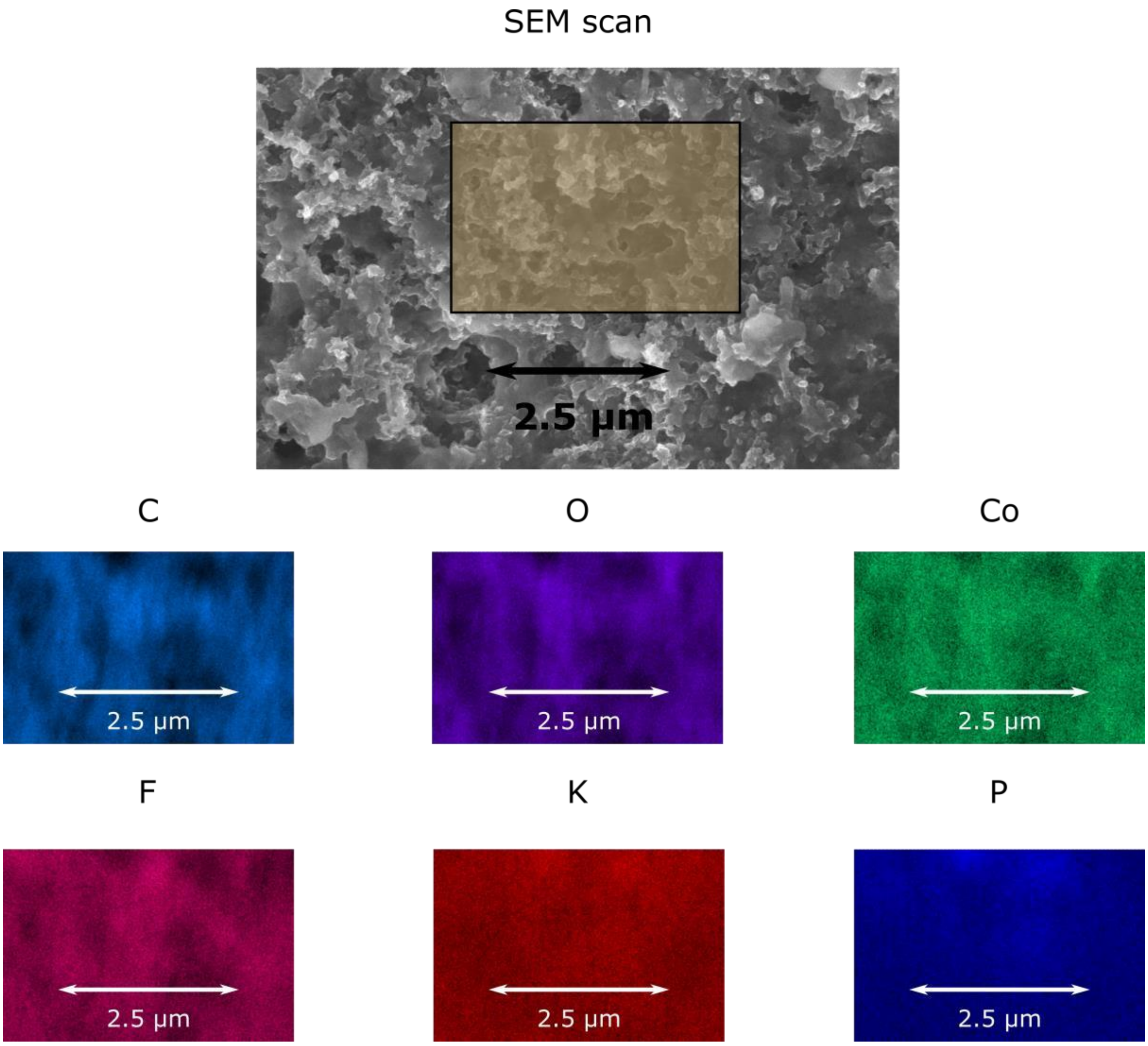

Fig. S11: EDXS intensity maps for JUMP-1 after electrochemical cycling at higher magnification.

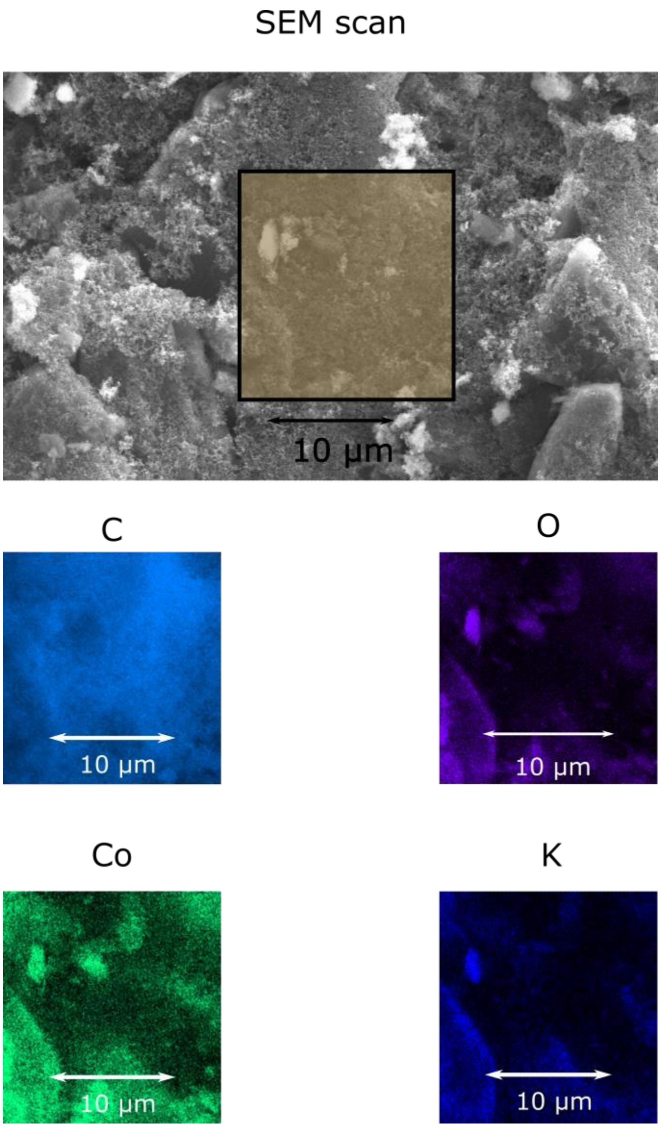

Fig. S12: EDXS intensity maps for JUMP-1(K) prior to electrochemical cycling at low magnification.

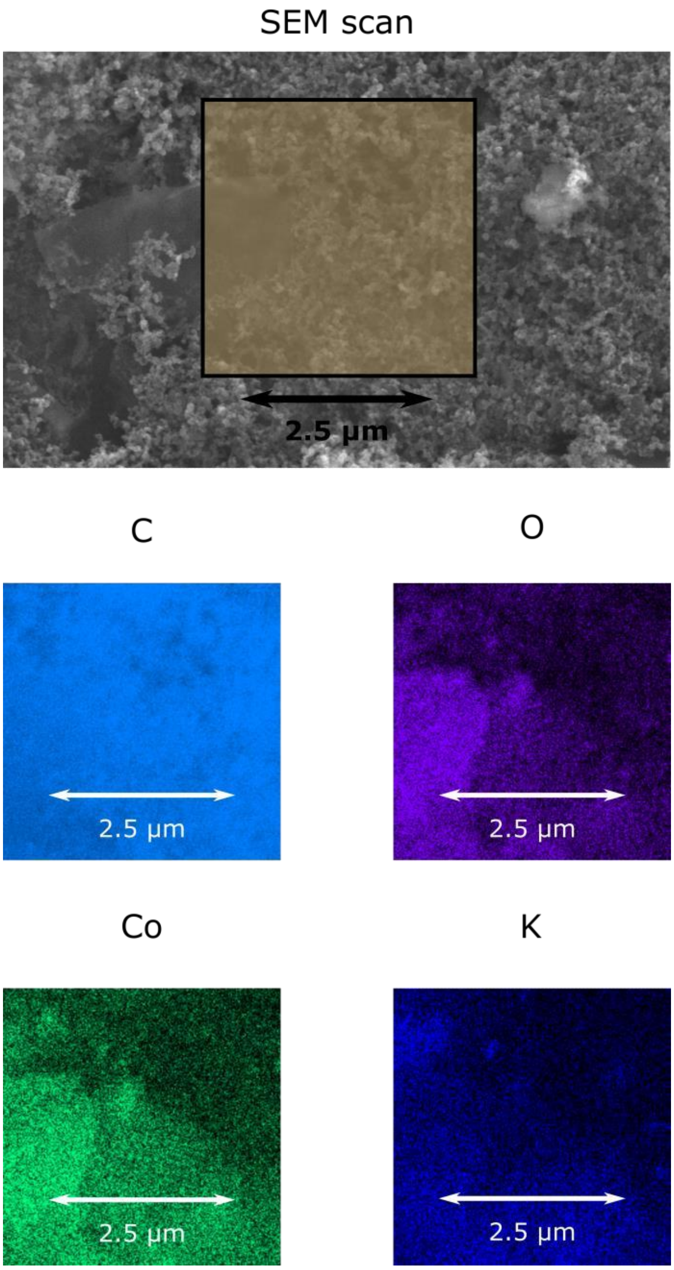

Fig. S13: EDXS intensity maps for JUMP-1(K) prior to electrochemical cycling at higher magnification.

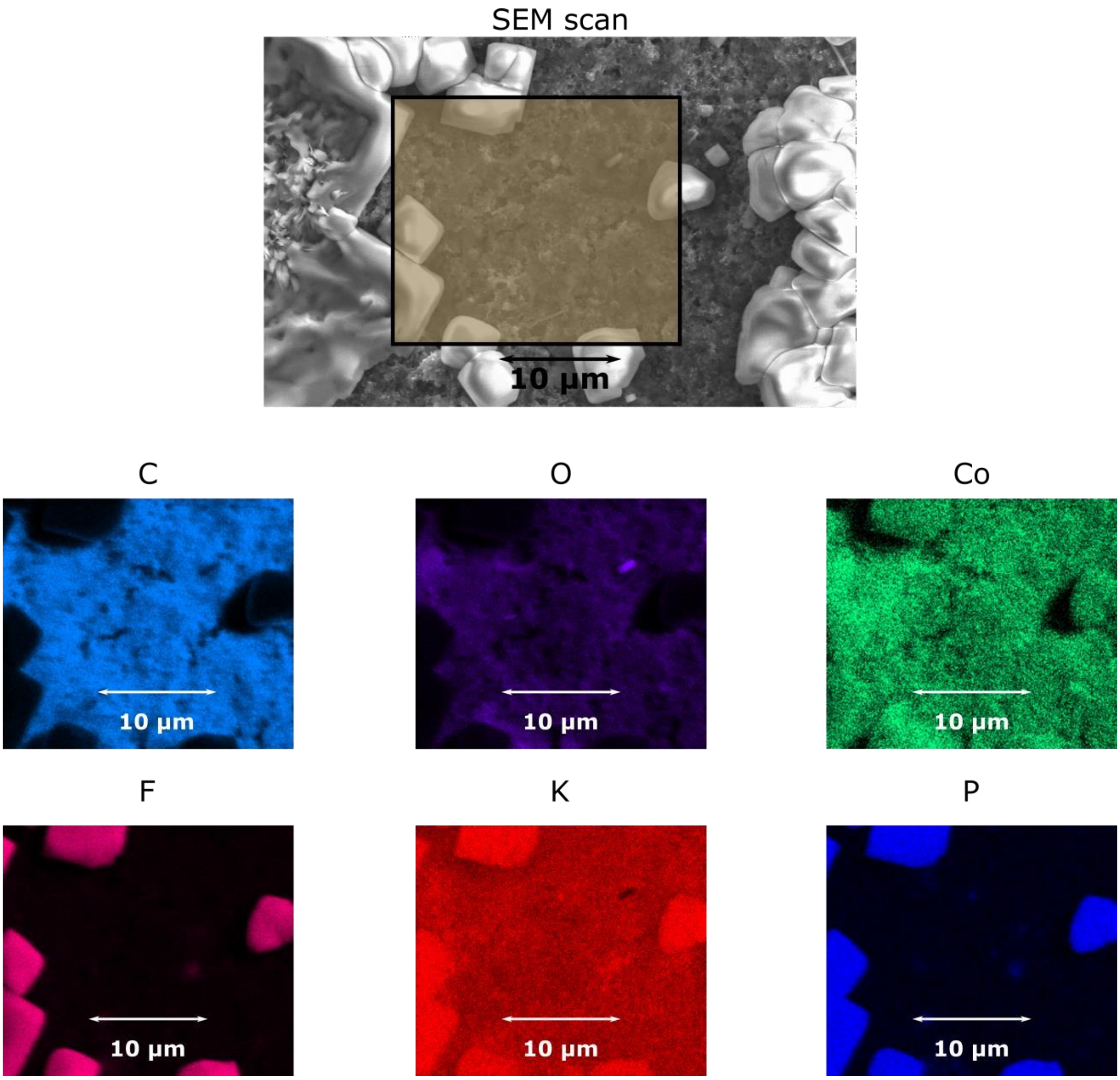

Fig. S14: EDXS intensity maps for JUMP-1(K) after electrochemical cycling at low magnification.

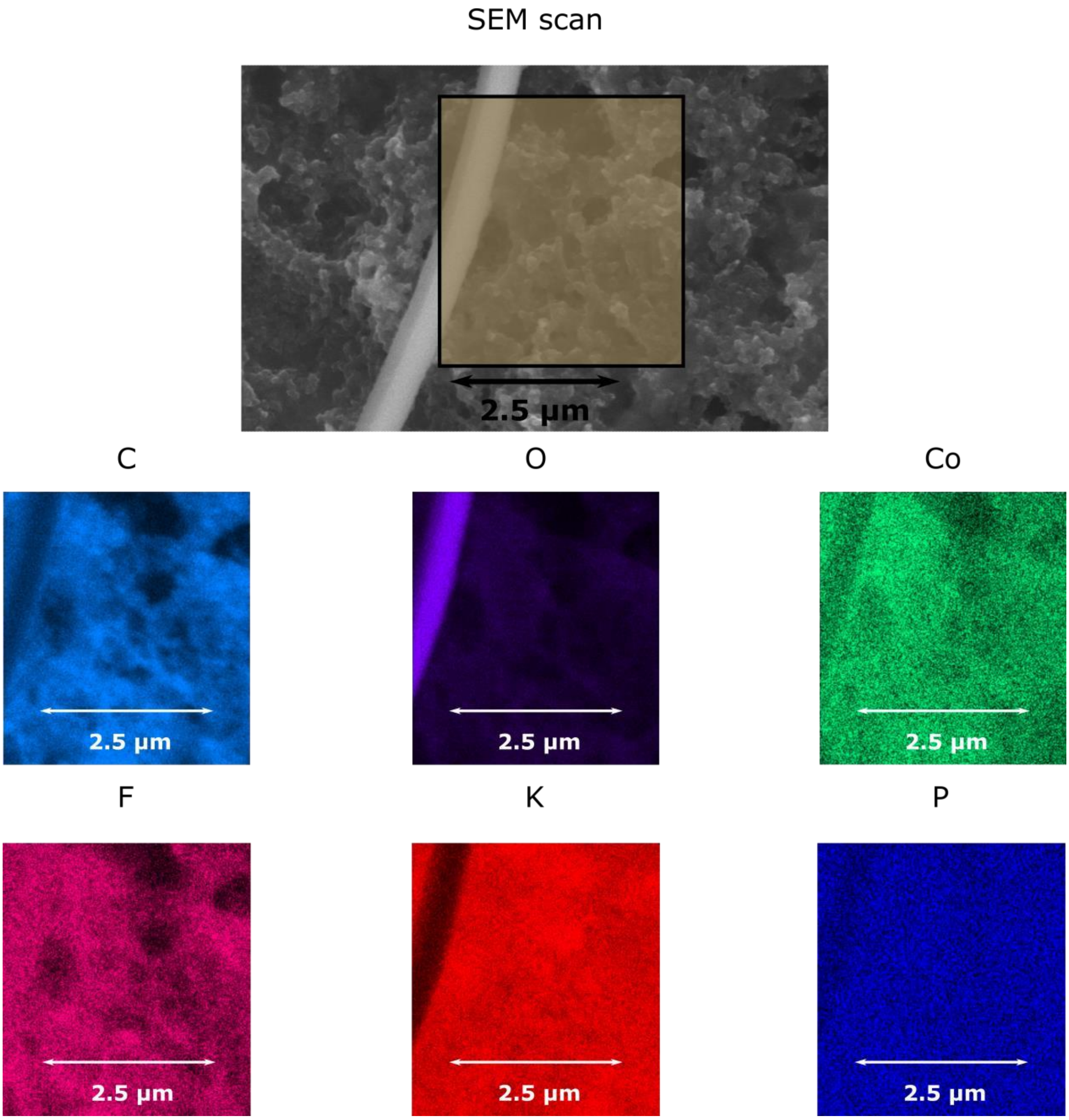

Fig. S15: EDXS intensity maps for JUMP-1(K) after electrochemical cycling at higher magnification.
